# Addressing the Need for a Better Understanding of Foot and Ankle Pathology Relevant to the Adolescent Athlete

**DOI:** 10.3390/children10081342

**Published:** 2023-08-03

**Authors:** Albert T. Anastasio, Samuel B. Adams

**Affiliations:** Department of Orthopaedic Surgery, Division of Foot and Ankle Surgery, Duke University, Durham, NC 27710, USA; samuel.adams@duke.edu

Foot and ankle pathology in the adolescent athlete is a growing area of basic science, translational, and clinical research. While pathologies such as glenohumeral internal rotation deficit (GIRD) [1] and anterior cruciate ligament (ACL) ruptures in high school-age throwing athletes, particularly in multi-sport female athletes [2], have been extensively studied, less has been explored on pathologies such as osteochondral lesion of the talus (OLT) [3] and ankle ligamentous injuries [4] in younger cohorts. Despite the relative focus on conditions proximal to the ankle, several advancements in recent years have substantially impacted our understanding and treatment of foot and ankle pathologies relevant to the adolescent athlete.

Regarding OLT, more focus has been placed on distinctions between youth presenting with this condition and adult cohorts [5]. Patel et al. demonstrated the poor utility of previously published criteria, which evaluate the stability of OLT on magnetic resonance imaging (MRI) in children. Instead, they proposed that regional skeletal maturity and older age were more predictive of unstable lesions [6]. Reilingh et al. present a series of 36 juvenile patients with OLT. These authors report a high failure rate of conservative treatment with good overall results from both chondral fixation and bone marrow stimulation [7]. Future work will continue to define treatment algorithms for adolescents with OLT and help define the long-term prognosis of the condition.

Ankle ligamentous injury is common in adolescents, and the presence of potentially open physes can substantially alter the specifics of the injury and the subsequent ramifications [8]. Even in younger children, recent research suggests that more than one-third of patients experienced recurrent sprains (average age of 9 years) [9]. Moreover, the presence of a fibular avulsion fracture has been associated with an increased risk of recurrent sprains. In seeking treatment, technologically savvy youth may be more likely to use social media websites to obtain information pertaining to ankle sprains and ankle injuries, and the quality of the content contained on these sites may be poor [10,11]. These and other pathologies remain relatively poorly described in the existing literature.

To address the paucity of literature related to foot and ankle pathology in the adolescent athlete relative to other orthopaedic pathologies and to advance our understanding of these conditions, we introduce a special series entitled “Foot and Ankle Pathology in the Adolescent Athletes” for the Journal *Children.* This series will accept manuscripts related to basic science, translational, and clinical aspects of foot and ankle care in adolescent athletes. World experts on the topic have been notified, and manuscript submission is ongoing.
